# Integrated *In Vitro*/*In Silico* Uncertainty Quantification Method for Protein Crystallization Models

**DOI:** 10.1021/acs.iecr.4c04517

**Published:** 2025-06-05

**Authors:** Daniele Pessina, Jorge Calderon De Anda, Claire Heffernan, Jerry Y. Y. Heng, Maria M. Papathanasiou

**Affiliations:** † Department of Chemical Engineering, 4615 Imperial College London, London SW7 2AZ, United Kingdom; ‡ The Sargent Centre for Process Systems Engineering, 4615 Imperial College London, London SW7 2AZ, United Kingdom; § Chemical Development, Pharmaceutical Technology & Development, Operations, 4978 AstraZeneca, Macclesfield SK10 2NA, United Kingdom; ∥ Institute for Molecular Science and Engineering, Department of Chemical Engineering, 4615 Imperial College London, London SW7 2AZ, United Kingdom

## Abstract

The complexity of protein crystallization and *in silico* modeling challenges process intensification and the wider adoption of crystallization in biomanufacturing. For computational models to support and replace extensive experiments, they must accurately reflect *in vitro* experiments. However, parameter estimation can be ineffective due to the highly nonlinear model structure and inaccurate online process analytical technology, which must be addressed. In this work, an experimentally validated and model-driven parametrization methodology is presented, developed for an antisolvent batch protein crystallization system with limited offline measurements. Global sensitivity analysis is performed to assess parameter identifiability during batch operations and inform optimal measurement points. Experiments at three different initial lysozyme concentrations (*c*_0_ = 15, 18, 19 mg/mL) are used for estimation. Parameter uncertainty distributions are recovered through an Approximate Bayesian Computation algorithm and propagated to model outputs through Monte Carlo simulations, avoiding linearization or unnecessary assumptions on the parametric and output uncertainty distributions. The methodology was successfully validated under two new experimental conditions. The shapes of the recovered parametric and output uncertainties highlight the need for parameter estimation methodologies specifically tailored to nonlinear models.

## Graphical abstract

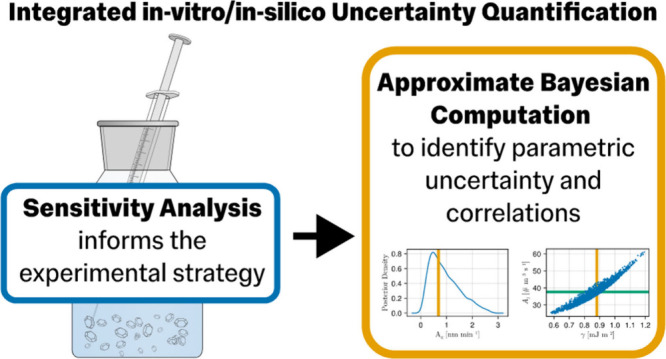

## 1 Introduction

The demand for advanced protein- and peptide-based therapeutics has grown considerably, with a projected market size of $566 billion USD by 2030.^1^ Following developments in the upstream processes that led to improved cell growth and product titers, the manufacturing bottleneck has shifted to the purification step, typically achieved via costly chromatographic separations.^2–4^ An alternative separation process in this space is crystallization, during which a highly pure and easily filterable crystalline solid of the target active pharmaceutical ingredient (API) is formed from solution. Crystallization is a promising and cost-effective alternative offering improved scale-up potential and final product form.^5^ Compared to an eluted liquid solution from a chromatographic column, the recovery of solid protein crystals offers improved thermodynamic stability, the possibility of long-term storage, and can enable the development of new therapeutic formulations.^6^ Wider adoption of crystallization is however challenged by the poor understanding of macromolecule crystallization and the complexity in identifying optimal operating conditions.^7^ For this, experimental “screens” are often used, in which conditions such as pH, protein, precipitant concentration, or temperature are systematically varied to examine how they alter the crystallization outcome. If a wide range of conditions is to be tested and if statistical confidence of the results is desired through multiple repeats of the same experiment, liquid volumes for each trial have to be reduced to prevent excessive API consumption and cost.^8^ Population-balance models (PBMs) can support and direct costly and time-consuming *in vitro* experimental campaigns by predicting the evolution of a crystallization process quicker and without any material cost. If *in vitro* and *in silico* efforts are integrated, process design and intensification can be accelerated to facilitate the adoption of crystallization in downstream pharmaceutical manufacturing.^9^

### 1.1 Opportunities and Challenges in *In Silico*/*In Vitro* Crystallization Approaches

#### 1.1.1 Parameter Identifiability and Estimation

Parameter estimation involves tuning kinetic parameters in order to fit model simulations to experimental measurements, obtaining a reliable PBM. The PBM’s mathematical structure impedes estimation, as it affects parameter identifiability; the parameters may be correlated or have unbalanced effects on model outputs, whereby some have a lower effect on model output than others and will be more difficult to identify.^10–13^ Fysikopoulos et al. presented a parameter estimability framework for multidimensional PBMs, combining orthogonalization of the local sensitivity matrix and a global sensitivity-based method to robustly determine the identifiable subset of 23 parameters from noisy measurements, and showed that the prediction accuracy can be improved if nonidentifiable parameters are instead fixed.^12^ Bhonsale et al. examined the parameter identifiability of air-jet mill PBMs (with a similar mathematical structure to crystallization PBMs) using a data-driven profile-likelihood approach and reported that they were able to fully identify the PBM only if measurements from a continuously operated mill were used.^10^ Additionally, fast breakage kinetics lowered the PBM’s identifiability, as fewer informative measurements could be used for parameter estimation. The aforementioned works show that complete identification of a PBM’s parameters may only be possible with specific equipment operation, high-frequency measurements, or may not be possible at all, and they highlight that identifiability should be assessed during model parametrization.

#### 1.1.2 Uncertainty Quantification

Parameter values typically carry uncertainty that can be calculated based on the measurements used for estimation. Bayesian frameworks consider both the probability of the observed data occurring and the prior belief of how the parameters are distributed to identify a probability distribution of each parameter. For example, Su et al. used Bayesian inference to successfully parametrize a reactive crystallization system in the presence of high-frequency *in situ* PAT probes and quantify its uncertainty.^14^ Due to the stiff and nonlinear model structure, parametric uncertainty quantification should not rely on assumptions that simplify the model structure at the expense of accurate quantification. These include, for example, normally distributed parametric uncertainty and linearization steps, which can lead to inaccurately identified elliptical parameter confidence regions and poorly predicting PBMs.^15^

Additionally, output uncertainty quantification steps should avoid linearization as discussed in the work of Bhonsale et al., in which linearization was shown to be less accurate than polynomial chaos expansion and Monte Carlo methods for uncertainty propagation.^16,17^ These challenges affect the parameter estimation’s effectiveness and should be tackled in the developed methodology by tailoring it to nonlinear and stiff model structures.

#### 1.1.3 Online Process Analytical Technologies for Protein Crystallization

Parametrization of a protein crystallization PBM is affected by inherent challenges stemming both from the mathematical structure and the quality and accuracy of the experimental measurements used for parametrization.^10,15^ However, there are obstacles that limit the number of available measurements. While modern Process Analytical Technology (PAT) probes have enabled accurate high-frequency, in situ measurements of solute concentration (and therefore supersaturation, the thermodynamic driving force behind crystallization) and the crystal size distribution (CSD) in many crystallization systems, PAT can sometimes fail at reporting accurate measurements of the crystallization batch in systems with sharp nucleation. For example, challenges in measuring lysozyme crystallization kinetics has been previously reported.^18^ Lysozyme has been widely used in the literature as a model protein, representative of general protein crystallization behavior, and it would be expected that experimental challenges observed in lysozyme systems would be found with other proteins as well.^19–29^ First, concentration tracking with an ATR-UV–vis spectrophotometer became inaccurate and noisy in the presence of small crystals.^18^ The authors observed that tiny crystals negatively affected *in situ* and offline UV–vis absorbance, and it was challenging to identify characterization peaks in the spectrum due to lysozyme’s large molecular weight and many bonds; the samples had to be drawn and centrifugated to mitigate the effect of small crystals. Second, due to the many and fine crystals nucleated, the authors also reported inaccurate measurements when using *in situ* particle-vision measurement (PVM) probes to measure the CSD throughout a batch. PVM measurement techniques generally rely on edge-detection algorithms to attempt to discern individual crystals from inline pictures of the reactor liquor. Due to the crystals moving in- and out-of-focus and rotating in the stirred solution, coupled with imperfect lighting conditions for edge-detection algorithms, calculating an average crystal size distribution can be inaccurate and makes PVM probes less reliable.

More robust and accurate measurements can instead be performed offline and therefore much less frequently. Protein concentration can be measured with a benchtop UV–vis spectrophotometer, and the CSD can be measured with a laser-diffraction particle size analyzer after filtration and drying of the broth. The need for offline measurement in turn affects parameter estimation, as fewer concentration measurements can be used in the loss function and the single possible CSD measurement dictates when the batch must be interrupted.

This work presents an integrated *in vitro*/*in silico* parameter estimation and uncertainty quantification methodology, specifically tailored to protein crystallization models. Sensitivity analysis is first used to inform parametric importance and measurement strategies for more effective parameter identification and estimation given the limited PAT accuracy and sparse available measurements. An Approximate Bayesian Computation method is used to recover the nonlinear PBM’s parameter confidence intervals while avoiding linearization and analytical functions of the likelihood. The identified parametric uncertainty is propagated to the outputs via Monte Carlo simulations. The methodology is applied to a batch antisolvent lysozyme crystallization system and validated experimentally.

## 2 Materials and Methods

The purpose of this work is to develop an uncertainty quantification methodology that leverages both experimental and computational tools to obtain an accurate PBM. Sensitivity analysis must first be carried out to inform the experimental strategy, after which lysozyme concentration and crystal size distribution measurements can be used for parameter estimation.

### 2.1 Experimental Protocol for Lysozyme Crystallization

Crystallization of hen-egg white lysozyme was performed in a stirred antisolvent batch crystallization setup at 20 °C, in a 0.1 M sodium acetate buffer (pH = 4.2) solution and with a 40 mL total solution volume per vessel. Sodium chloride (NaCl) was used as the precipitating agent (50 mg/mL postdilution). Lysozyme was dissolved at 5 different concentrations (*c*_0_ = 15, 18, 19 mg/mL for parameter estimation, *c*_0_ = 16, 20 mg/mL for parameter validation) and gently stirred until all powder had dissolved. Each protein solution was then filtered with a 0.22 μm filter and stored in an incubator at 30 °C until use. Precipitant solutions were prepared by dissolving NaCl (100 mg/mL) in the buffer solution and gently agitating until fully dissolved. Experiments used for parameter estimation were repeated twice (total of 3 experiments at each condition).

Crystallization was carried out in three capped 100 mL glass flasks, where 20 mL of the protein and precipitant solutions, respectively, was in each flask and placed in a water bath set to 20 °C. The crystallization solution was gently stirred with small magnetic stirring bars at 100 rpm to ensure that spatial variation of the lysozyme concentration within the solution can be ignored. A workflow of the experimental procedure is given in Figure 1.

**Figure 1:**
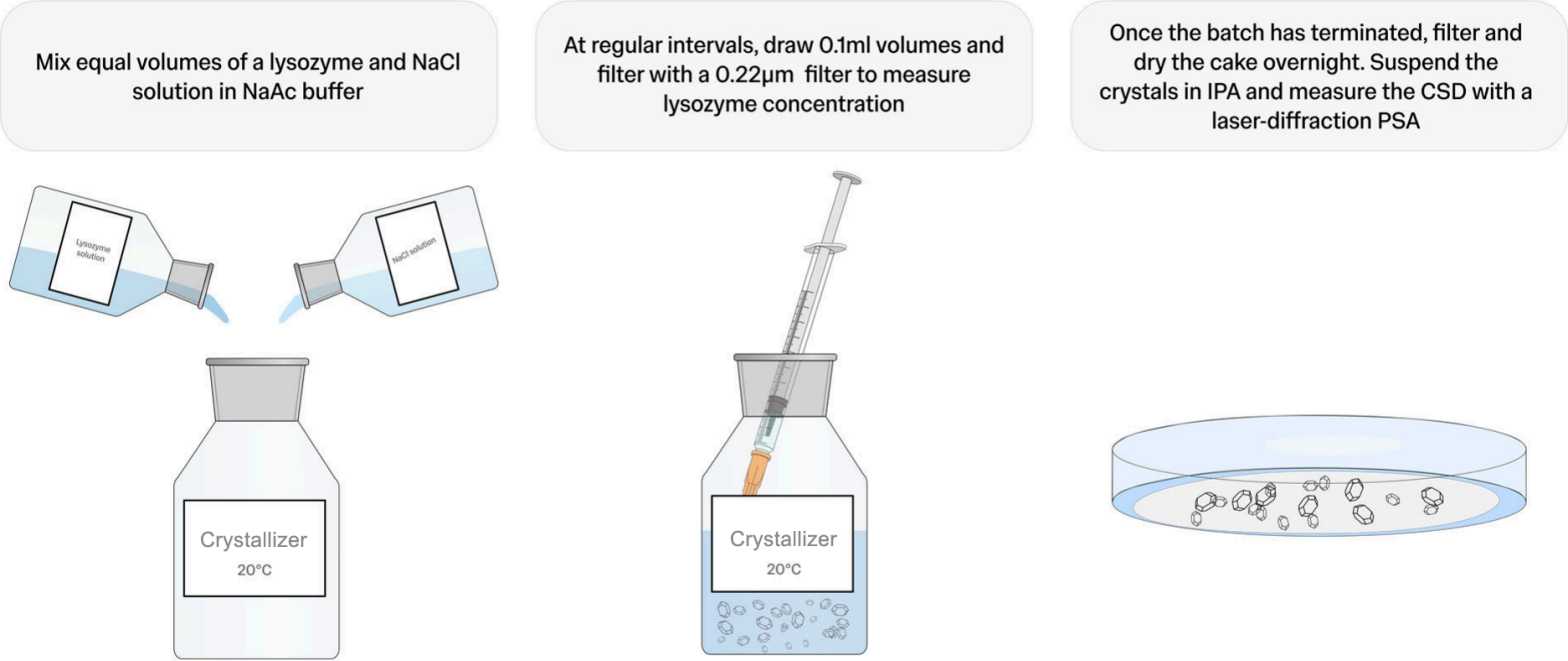
Workflow of the crystallization experiments performed.

Lysozyme concentration measurements were performed approximately every 30 min with a Thermo Fisher NanoDrop One UV–vis spectrophotometer unit (extinction coefficient = 26.4 at 280 nm for a 1% lysozyme solution). For each concentration measurement, approximately 0.1 mL was drawn from solution and filtered with a small 0.22 μm syringe filter to trap the solids, avoiding centrifugation and the risk of crystal breakage and dissolution. In this way, the fine crystals were ensured to not affect the UV absorbance or raise the solute concentration.

At the batch completion, the solution was filtered with a vacuum pump and dried overnight. The collected solids were suspended in isopropyl alcohol to prevent their dissolution and ultrasonicated to separate the dried crystals. The CSD was measured with an Anton Paar PSA 1190 laser-diffraction particle size analyzer, with further stirring and sonication of the solids in the sample tank during the size measurements.

### 2.2 Computational Methodology

A workflow of the developed integrated *in silico*/*in vitro* methodology for parameter estimation and uncertainty quantification is shown in Figure 2. The first step involves global sensitivity analysis performed before experimental measurements to inform optimal measurement times. Following crystallization experiments, optimization routines explore the parameter space to fit model solutions to the measurements and quantify the parametric uncertainty, which is then propagated to the model outputs via Monte Carlo simulations to quantify the output uncertainty.

**Figure 2:**
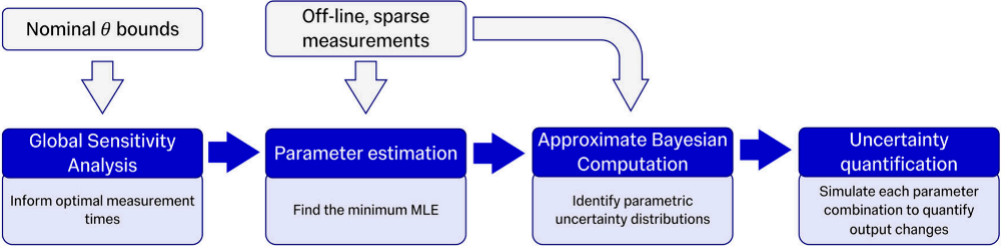
Workflow for the presented parameter estimation and uncertainty quantification methodology.

#### 2.2.1 PBM Formulation and Solution

The PBMs presented were formulated with a one-dimensional population-balance equation for a batch crystallizer with primary homogeneous nucleation rate *B*_0_, size-independent growth *G*, and negligible aggregation and breakage (eq 1), with the respective initial (eq 2) and boundary (eq 3) conditions. The same assumptions have been used in published works to successfully model lysozyme and other crystallization systems.^14,29–31^ The supersaturation ratio (eq 4), the ratio of solute concentration in solution and at saturation, is the thermodynamic driving force of the crystallization process. Since the population-balance is a hyperbolic partial-differential equation, the equations were discretized following a high-resolution finite-volume scheme from Qamar et al.^32^ A mass balance is included in the PBM, accounting for solute consumption via crystallization during the batch (eq 5), and energy balance can be omitted since the crystallizer is operated isothermally. Each semidiscrete PBM was formulated with a 300-bin domain (*L*_*min*_ = 1 nm, *L*_*max*_ = 50 μm), Superbee flux limiter, rel. tol. = 10^–4^, abs. tol. = 10^–6^. The resulting set of ODEs was solved using the *Julia* coding language employing an explicit Strong-Stability Preserving Runge–Kutta time-stepping method.^33,34^

$$\frac{\partial n}{\partial t}+\frac{\partial \left(Gn\right)}{\partial L}=B_{0}\delta \left(L-L_{min}\right)$$ 1

n(L,t=0)=0 2

$$n\left(L_{\min},t\right)=B_{0}\left(S\left(t\right)\right)$$ 3

$$S=c\left(t\right)/c_{\mathrm{sat}.}$$ 4

$$\frac{dc}{dt}=-3\rho_{c}k_{v}\int_{0}^{\infty }GnL^{2}\;dL$$ 5

The presented PBMs were formulated with classical nucleation theory (CNT) nucleation (eq 6) and empirical growth kinetics (eq 7).^35^ The parameters to be estimated are the pre-exponential nucleation constant *A*_*j*_, surface energy γ, pre-exponential growth constant *A*_*g*_, and growth exponent *g*, bold-faced in eqs 6 and 7. Note that a log transformation is applied to *A*_*j*_ to scale the parameter’s magnitude to be similar to those of the other regressed parameters. Other physical parameters, such as *k*_*v*_, ρ_*c*_, and *v*_0_, are assumed to be well-known. The values used in this work have been collected in Table 1.

$$\mathrm{CNT:}\;B_{0}=\exp A_{j}S\;\exp\left(\frac{-16\pi \gamma^{3}v_{0}^{2}}{3{k_{B}}^{3}T^{3}\;\ln^{2}S}\right)$$ 6

$$\mathrm{Emp.:}\;G=A_{g}{\left(S-1\right)}^{g}$$ 7

**Table 1:** Collected Model Parameters

Parameter	Value	Reference
*k*_*v*_	0.81	Mitchell et al., 2023 ^29^
ρ_*c*_	1240 kg m^–3^	Leung et al., 1999 ^36^
*v*_0_	2.97 × 10^–26^ m^–3^	Nadarajah et al., 1996 ^37^
*A*_*j*_	Fitted during parameter estimation	
γ	Fitted during parameter estimation	
*A*_*g*_	Fitted during parameter estimation	
*g*	Fitted during parameter estimation	

#### 2.2.2 Global Sensitivity Analysis

Global sensitivity analysis (GSA) methods quantify the effect of model parameters on the output uncertainty and can be used to find the degree to which model outputs are sensitive to changes in input parameters. Time-indexed sensitivity indices for each kinetic parameter are calculated and used to rank parameters based on their identifiability.^38,39^ Importantly, GSA is not used to identify a reduced-order model but rather to assess the model identifiability. If this step precedes *in vitro* experiments, the evolution of sensitivity indices between different outputs and across the batch can be an indicator of favorable measurement times to ensure all parameters are as identifiable as possible.^40^

#### 2.2.3 Parameter Estimation via Single-Objective Optimization

Parameter estimation is posed as a single-objective optimization problem in which a single combination of kinetic parameters can be found by minimizing the maximum likelihood estimator (MLE) loss function (eq 8). The loss function includes the squared model-measurement misprediction of both the concentration trajectory and final 50% quantile *D*_50_ and the experimental measurement variances σ_*O*,*e*,*m*_^2^ (output *O*, experiment *e*, and measurement *m*) to scale each deviation by the experimental uncertainty. Each repeat’s experimentally measured concentration is used to calculate the average *c*_*avg. measured,m*_ and estimate the variance σ_*O*,*e*,*m*_^2^. By including the variance in the loss function, the prediction of low experimental-variance points during the batch is effectively prioritized over more experimentally uncertain regions by the MLE since the estimated variances are nonconstant throughout batch operation. A gradient-free DE genetic algorithm was used to find the minima.^41^

$$\mathrm{MLE}\left(\theta \right)=\overset{N_{\mathrm{exper}.}}{\underset{e=1}{\sum }}\left[\overset{N_{\mathrm{conc}.,e}}{\underset{m=1}{\sum }}\frac{{\left(c_{\mathrm{avg. measured},m}-c_{\mathrm{model}}\left(t=t_{m},|\theta \right)\right)}^{2}}{\sigma_{\mathrm{conc}.,e,m}^{2}}+\frac{({D_{50}}_{\mathrm{avg. measured},\mathrm{end}}-{D_{50}}_{\mathrm{model}}\left(t=t_{\mathrm{end}}|\theta \right){)}^{2}}{\sigma_{D_{50},e}^{2}}\right]$$ 8

#### 2.2.4 Identification of the Confidence Region

Parametric and output uncertainties can be assessed by introducing confidence intervals and regions after the most-optimal set of kinetic parameters has been found by the single-objective search algorithm. Confidence intervals of the estimated parameters can be approximated via calculation of the Hessian matrix of the log-likelihood loss function evaluated at the optimal parameters θ*_opt_*, therefore requiring an analytical and twice-differentiable equation for the likelihood.^42,43^ This route is incompatible with a black- or gray-box model from which it is challenging or impossible to calculate first- and higher-order derivatives. Instead, a confidence region can be defined and extracted from point-wise simulations of candidate parameters θ and the corresponding MLE­(θ), thereby avoiding linearization. Equation 9 gives an upper-bound value of the loss function following the Fisher distribution, confidence level α, *p* parameters, and ν – *p* degrees of freedom. By perturbing parameter combinations, simulating the model, and computing the loss function, a statistical representation of the confidence region and parameter distributions can be recovered, assuming the measurement errors are normally distributed.^44^ Since pooled measurements from the experimental repeats are used to estimate the variances in eq 8, ν corresponds to the number of experimental measurements used in the loss function. The Fisher distribution is used in eq 9 rather than a χ^2^ distribution because the error variances are estimated from a limited number of measured data points. With appropriate scaling and approaching an infinite number of measurements, the Fisher distribution becomes equivalent to the χ^2^ distribution. It is also expected that the number of measured concentrations will be greater than the number of average crystal size measurements since the latter can only be measured once at batch termination due to experimental restrictions. In eqs 8 and 9, the concentration misprediction is not averaged by the number of concentration measurements. Rather, each misprediction is summed and added to the average crystal size distribution under the assumption that the ratio of estimated variances is known.

$$\mathrm{MLE}\left(\theta \right)<\mathrm{MLE}\left(\theta_{\mathrm{opt}}\right)\left(1+\frac{p}{\nu -p}F_{p,n-p}^{\alpha }\right)$$ 9

Approximate Bayesian Computation (ABC) offers likelihood-free methods to recover the parameter posterior distribution in cases where the likelihood function is intractable, for example, if the simulator platform is treated as a black-box and if simulation costs are high.^45^ In ABC methods, a maximum deviation from the target likelihood is set. Next, parameter candidates are drawn from an initial parameter prior distribution and simulated, from which the computed MLE loss function values can be either accepted or rejected. With enough coverage of points inside the confidence region, approximate parametric uncertainty distributions can be recovered and propagated with Monte Carlo simulations to quantify the prediction uncertainty, avoiding linearization. In this work, the ABC with Differential Evolution (ABCDE) method from Turner and Sederberg was implemented in *Julia*.^46^ Further details on ABC and ABCDE methods can be found in the Supporting Information.

#### 2.2.5 Uncertainty Propagation to Model Outputs

Lastly, the collected population of parameter values serves as a source of uncertainty to be propagated. Output confidence intervals are quantified by simulating every parameter candidate and collapsing the concentration and quantile trajectories into distributions of possible values. Gaussian error is assumed only for experimental measurements, while the remaining steps to find both parameter and output confidence intervals are purely model-driven, thereby accounting for the model’s stiffness and nonlinearity when quantifying the uncertainty.

## 3 Results and Discussion

### 3.1 Evolution of Sensitivity Indices during Batch Operations

In the first step of the methodology, a variance-based GSA was performed around the collected nominal values (Table 2). These nominal values, collected from published literature of lysozyme crystallization models, indicate plausible true values of the parameters around which to perform GSA. Given the lack of published lysozyme models parametrized against data from the same experimental conditions used in this study, some of the values come from studies with similar but distinct experimental conditions. From a quasi-random Sobol’ sequence, 8192 combinations of kinetic parameters were generated and simulated. Concentration and quantile trajectories were then tabulated and passed to the SobolGSA software, which calculates first-, second-, and total-order sensitivity indices with a Random Sampling High Dimensional Model Representation (RS-HDMR) supporting surrogate.^47^ The interested reader is referred to the Supporting Information and publications by Saltelli et al. and Kucherenko for further details on GSA and the RS-HDMR surrogate.^47,48^

**Table 2:** Parameter Bounds Used for GSA and Associated References

Parameter	*A*_*j*_ [log # m^–3^ s^–1^]	Ref	γ [mJ m^–2^]	Ref	*A*_*g*_ [nm min^–1^]	Ref	*g* [−]	Ref
Lower bound	22.7	Lin et al., 2017 ^26^	0.32	Lin et al., 2017 ^26^	0.394	Mitchell et al., 2023 ^29^	2	-
Upper bound	32.9	Mitchell et al., 2023 ^29^	1.0	Galkin and Vekilov, 2000 ^21^	0.876	Pusey et al., 1986 ^49^	3	-

Figure 3 shows the evolution of first and second sensitivity indices across the batch time for both the solute concentration *c*(*t*) and the 50% quantile *D*_50_(*t*), with total-order sensitivity indices shown in Figure 4. Sensitivity indices for other quantiles were identical to the ones shown, as the model did not include aggregation or breakage kinetics. A cutoff of *s*_*T*_ < 5% was set to reflect the expected experimental error, beyond which parameters were considered inestimable, as their effects would not be discernible from *in vitro* experiments.

**Figure 3:**
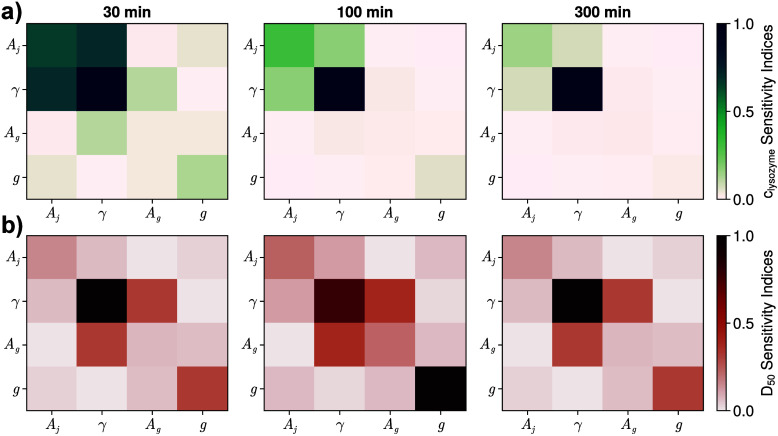
Sensitivity index heatmaps for (a) concentration and (b) 50% size quantile model outputs at 3 different points of the batch.

**Figure 4:**
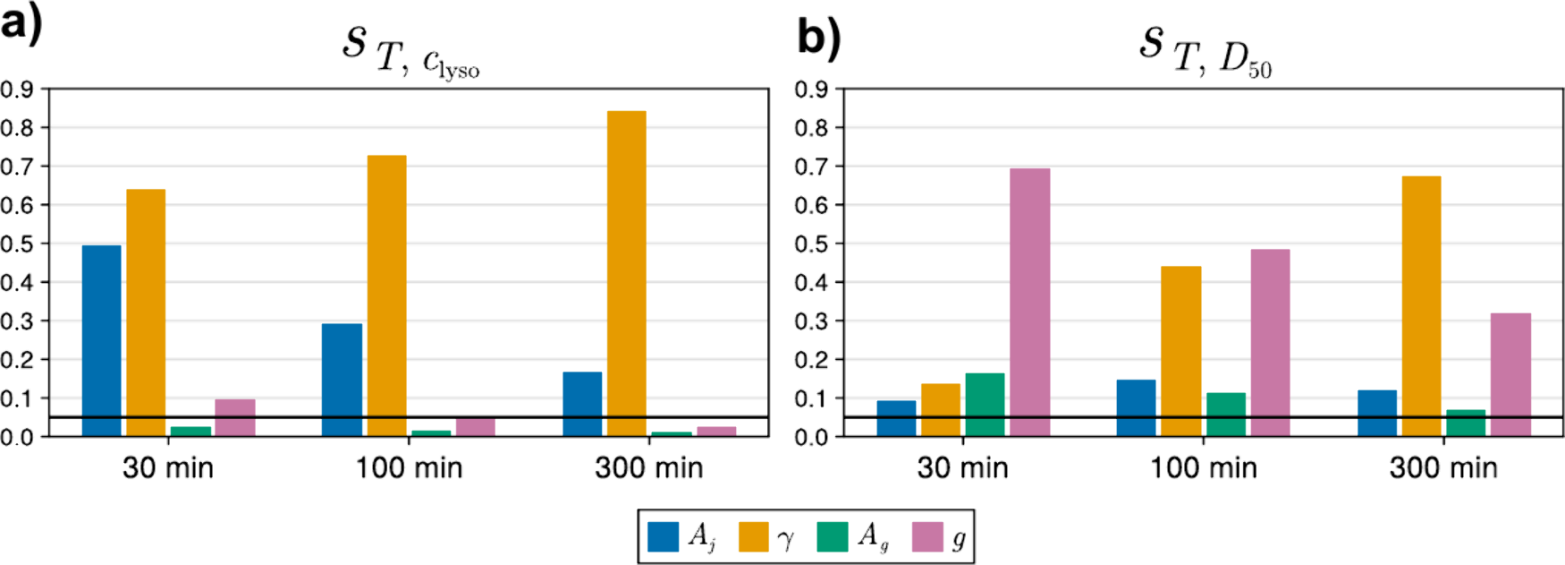
Evolution of total-order sensitivity indices for (a) concentration and (b) 50% size quantile model outputs at 3 different points of the batch. The black line represents the 5% experimental error cutoff.

Single- and total-order indices for lysozyme concentration (Figure 4a) show that the surface energy γ dominates over the others and increases in significance as the batch progresses, indicating that the parameter is expected to be well-identifiable, while indices for all other parameters instead decrease. *A*_*j*_ remains moderately significant and identifiable as the indices approach the experimental error cutoff during batch operation. In contrast, the Sobol’ indices for the growth parameters *A*_*g*_ and *g* are lower than 10% early in the batch, and the growth kinetics quickly become unidentifiable from the concentration measurements alone. Nucleation kinetics are instead more significant and easier to identify, suggesting that lysozyme consumption in solution is nucleation-dominated. The second-order indices also support this statement, as the strongest cross-parameter interactions are between the two nucleation parameters.

Sensitivity indices for the *D*_50_ quantile (Figure 4b) are noticeably different. The surface energy γ again has increasing sensitivity indices during the batch. However, *A*_*g*_ and *g* are more significant for the *D*_50_ output; at 30 min into crystallizer operation, the growth exponent *g* is the most identifiable parameter, and both growth parameters remain identifiable at 300 min of residence time. This result can be explained by the unseeded (crystal-free) initial condition. Early in the batch, few crystals have nucleated, and nucleation has not occurred long enough to affect the quantiles whose change would mainly be growth-driven. As the batch progresses, a higher concentration of crystals is present in solution, and the total amount of nucleation occurring since the batch start is much greater; therefore, a higher number of individual crystals must grow to increase the quantile. The decreasing sensitivity of *A*_*g*_ and *g* indicates the parameters will eventually become unidentifiable, suggesting that long batches should be avoided if all parameters are to be estimated.

The different sensitivity profiles between the two model outputs *c*(*t*) and *D*_50_(*t*) inform the two critical points of the experimental strategy. First, sole measurement of solute concentration is not enough to identify all four kinetic parameters, and the CSD should be measured too. Second, given the decreasing identifiability of *A*_*g*_ and *g* over time, the batch should not be excessively long. In practice, since only a single offline particle size measurement can be performed, and only after batch interruption, the crystals must have grown enough to be above the particle size analyzer’s detectable limit.

### 3.2 Identification of Parameter Posterior Distributions

The experimental data used for parameter estimation and model validation, which include measured lysozyme concentrations, measured size distributions, *D*_50_, and the respective estimated variances, are collected in the Supporting Information. The parameter search bounds for the single-objective optimization routine are presented in Table 3. Since the initial optimization routines returned solutions on the initial parameter bounds in Table 2, the search bounds were widened to assess the PBM’s predictive capability. The differential evolution optimization problem was set with 512 particles, 512 generations, step size = 0.7, and cross-over = 0.5. Multiple runs of the differential evolution optimizer, along with optimization results using gradient-based optimizers, all returned the same optimal parameters.

**Table 3:** Parameter Search Bounds for the DE Algorithm

Parameter	*A_j_* [log # m^–3^ s^–1^)]	γ [mJ m^–2^]	*A*_*g*_ [nm min^–1^]	*g* [−]
Lower bound	10	0.15	0.15	1
Upper bound	60	1.2	2.5	4

The differential evolution algorithm found the optimal parameter vector to minimize measurement-model mispredictions (Table 4). The optimal parameters also suggest that the estimation was successful, as all except *A*_*j*_ are within the literature values used for GSA (Table 2), and the pre-exponential constant, which is a kinetic parameter rather than a thermodynamic one, remains on the same order of magnitude as the reported values. After identification of the minimum MLE achievable from the chosen model formulation, an MLE upper bound for the 95% confidence region was calculated from eq 9 to be used for posterior identification and uncertainty quantification.

**Table 4:** Optimal Parameters and ABCDE-Derived Parameter Posterior Distributions

Parameter	*A*_*j*_ [log # m^–3^ s^–1^]			γ [mJ m^–2^]			*A*_*g*_ [nm min^–1^]			*g* [−]		
Optimal parameters	37.7			0.881			0.682			2.40		
16%, 50%, 84% posterior quantiles	30.7	36.7	43.4	0.739	0.881	0.967	0.379	0.819	1.56	1.96	2.26	2.63
MLE_min_				193.2			MLE_95% CI_			287.2		

The optimal MLE was scaled to find the MLE upper bound, which defines the confidence region following eq 9 and the chosen confidence level. The degrees of freedom were calculated by summing the number of individual measurements included in the loss function ( $\overset{N_{\mathrm{exper}.}}{\underset{e=1}{\sum }}$ ­[ $\overset{N_{\mathrm{conc}.}}{\underset{m=1}{\sum }}$ + 1]) and subtracting the number of estimated parameters. The upper MLE bound was set as the ABCDE’s target, such that any point between it and the minimum is considered as inside the region. Parameter prior distributions must be defined to initialize the first generation of candidates. To avoid any assumptions on the parameter’s distributions, each prior was defined as a uniform rectangular distribution bounded by the same search bounds used in the initial optimization routine. 4096 points were used in each ABCDE generation to smoothly approximate the parameter posteriors. After the first sampling from the parameter priors, the ABCDE procedure was continued for 128 generations in which the population is iteratively refined and improved until each of the 4096 points is inside the confidence region. The choice of prior method only has a minor effect on the computational expenditure of the method. Since candidate parameters are sampled from the prior only once, after which the method continues with differential evolution iterations, the computational expenditure is dictated by how quickly the differential evolution algorithm converges to the credible region. In this work, differential evolution was found to be robust, converging to the local minima (in the case of single-objective optimization) and to the credible region (in the case of ABCDE) in approximately 100 generations with the parametric bounds given in Table 3.

Once ABCDE had terminated, a 4096-row table of parameter combinations and their corresponding MLE were collected from which parameter distributions can be recovered. The parameter combinations were collapsed into arbitrary distributions and plotted into a corner or pair plot.

Figure 5 shows a corner plot of the final ABCDE generation, with optimal parameter values overlaid. Parameter distribution properties are also collected in Table 4. The diagonal plots (a–e) show each parameter’s uncertainty distribution in the confidence region, with the uniform prior distributions also shown. The MLE profiles for each parameter are shown in subplots (l–o), noting that the optimal parameter lines intersect with the MLE minima. The parameters *A*_*j*_, γ, and *g* appear to be normally distributed and approximately centered around the optimal combination θ*_opt_*. Since *A*_*g*_ is physically bounded to values greater than 0, it instead presents a more skewed distribution, which asymmetrically extends to larger values while remaining in the credible region. This indicates that the assumption of normally distributed parameter uncertainty would not have been suitable.

**Figure 5:**
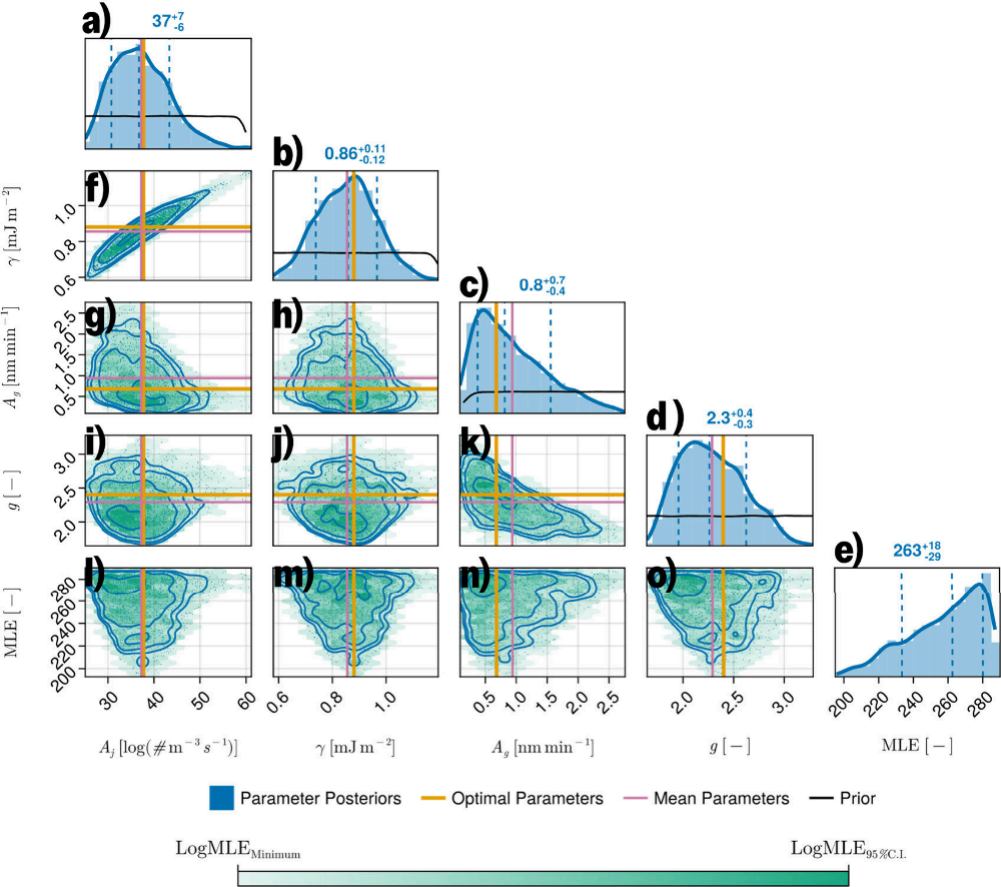
Corner plot of the final ABCDE generation with 4096 points. Outer plots (a–e) show each parameter’s distribution. The values shown correspond to the 50%, 16%, and 84% quantiles of each distribution. Interior plots (f–o) contain scatter points of each point and contours corresponding to standard deviations from the mean. Orange lines correspond to the optimal parameter values found in the first optimization routine. Pink lines correspond to the 50% quantile of each distribution. Black lines correspond to the prior distribution.

The interior plots (f–o) instead show cross-parameter correlations (or lack thereof), with Spearman rank correlation coefficients reported in Table 5. A quasi-linear correlation between *A*_*j*_ and γ can be observed in Figure 5f, confirmed by the high correlation coefficient. A more triangular-looking correlation between *A*_*g*_ and *g* can also be observed in Figure 5k. These correlations are not particularly unexpected, as the parameters in each pair belong to the same kinetic equation. Other combinations of parameter pairs do not display large amounts of correlation, with γ × *A*_*g*_ and γ × *g* having almost-zero correlation coefficients. The nonelliptical shapes of the reconstructed confidence regions again highlight the strengths of the developed methodology in accounting for the PBM’s nonlinearity, as linearization would have yielded incorrect elliptical regions and a mispredictive parametrized PBM.

**Table 5:** Spearman Correlation Coefficients for Each Pair of Parameters

Parameter Combination	*A*_*j*_ × γ	*A*_*j*_ × *A*_*g*_	*A*_*j*_ × *g*	γ × *A*_*g*_	γ × *g*	*A*_*g*_ × *g*
Spearman correlation coefficient	0.968	–0.356	–0.019	–0.207	–0.012	–0.680

### 3.3 Output Uncertainty Quantification

Figure 6a,b illustrates the model performance and how this compares to experimental measurements. Specifically, Figure 6a contains model predictions of the experimental data used for parameter estimation, with the estimated *D*_50_ quantile in the legend label, while Figure 6b instead contains model predictions of the experiments used for validation. The measured and predicted quantiles are also collected in Table 6. Examining the fit to the measurements used for parameter estimation (Figure 6a), there is good prediction of concentration profiles of the two experiments with lower initial concentrations (*c*_0_ = 15, 18 mg/mL) and poorer prediction of the experiment with high initial concentration. There is also misprediction between the predicted and measured *D*_50_ quantile values except for the experiment with *c*_0_ = 15 mg/mL. Regardless of the imperfect fit to the estimation measurements, from the validation simulations, it appears that the parameter estimation procedure was successful (mean absolute error: 0.748 mg/mL, 6.76 μm). Lysozyme concentration predictions and uncertainty bands are in good agreement with the experimentally measured points. The *D*_50_ quantile prediction is less accurate, especially for the optimal parameter trajectory. The *D*_50_ uncertainties are quite wide, suggesting that the parameter estimation could be improved by further size measurements during the batch. The model mispredictions could be accredited to the use of inaccurate, simplifying assumptions of the population-balance equation (eq 1) or to the choice of kinetic expressions for crystal nucleation and growth. First, negligible breakage and agglomeration of the crystals is assumed. While microscope pictures did not indicate the presence of any of the two phenomena, inaccessible *in situ* PAT measurements make it challenging to confirm that aggregation and breakage do not occur throughout the whole batch duration. It appears that it is also possible that the lysozyme crystals nucleate and grow through multiple mechanisms which cannot be solely explained by CNT and power-law growth. However, introducing multiple kinetic expressions also introduces more kinetic parameters, which likely negatively affect the model’s identifiability in the context of scarce experimental measurements.

**Table 6:** Measured and Predicted *D*_50_ Quantiles of the Estimation and Validation Experiments

Experiment	Estimation Exp. 1	Estimation Exp. 2	Estimation Exp. 3	Validation Exp. 1	Validation Exp. 2
Measured *D*_50_ [μm]	10.73 ± 2.3	9.39 ± 2.0	7.26 ± 1.0	13.73	11.13
Optimal *D*_50_ [μm]	4.78	5.59	8.64	7.3	4.22
UQ *D*_50_ [μm]	5.17 ± 2.2	5.96 ± 2.4	8.72 ± 3.4	7.59 ± 2.9	4.65 ± 2.1

**Figure 6:**
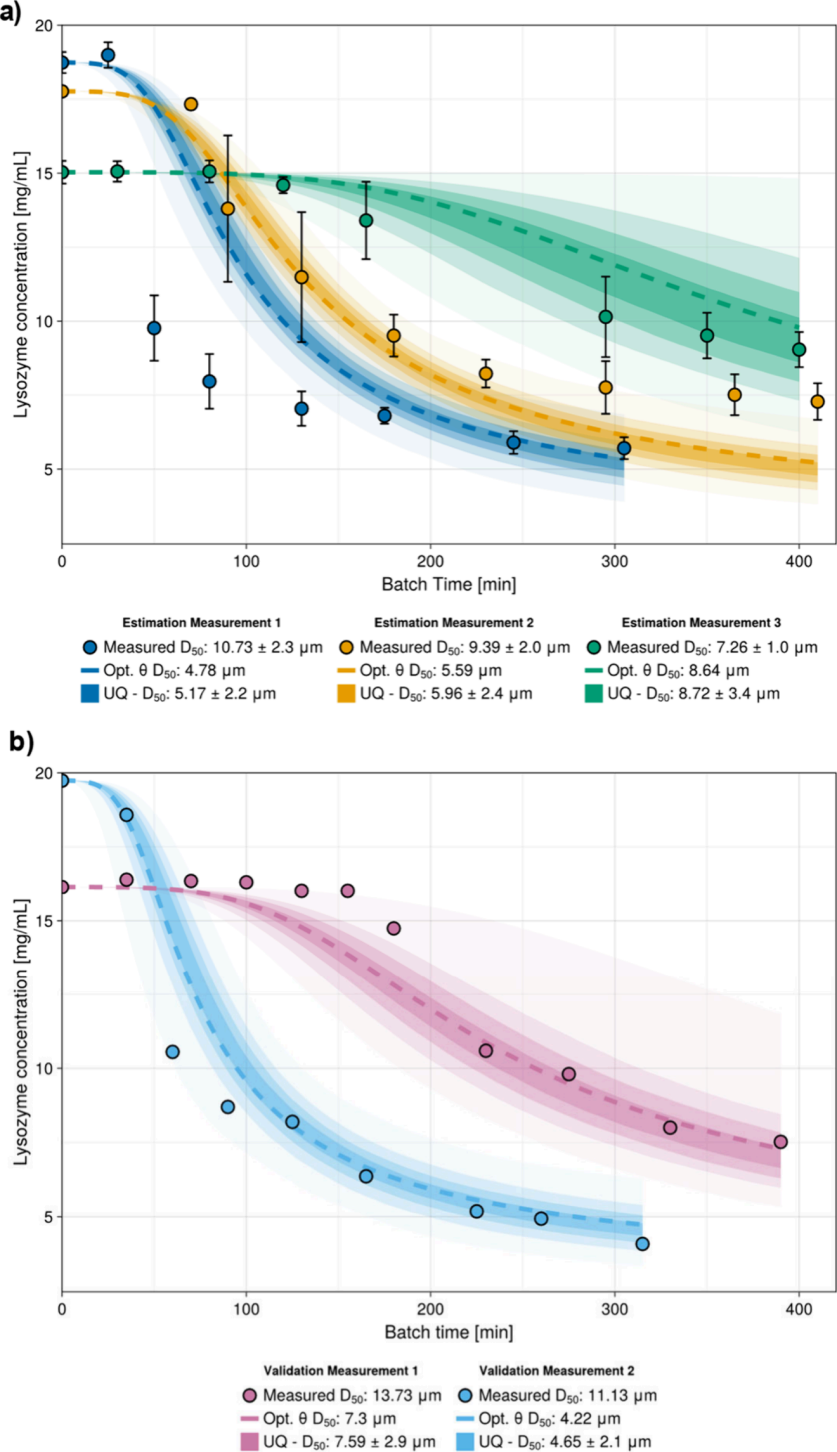
Simulations showing prediction and UQ of the measurements used for (a) parameter estimation and (b) validation of the model. Shaded regions define the centered 25%, 50%, and 75% and full regions of the collected trajectories for the 95% confidence interval.

The most significant model misprediction of the measurements used for parameter estimation is found in the prediction of “Estimation Measurement 1”, which was carried out with *c*_0_ = 19 mg/mL. The model error could be attributed to the high initial supersaturation. At such conditions, both nucleation and growth progress very quickly, and multiple nucleation and growth mechanisms may be occurring simultaneously which do not occur at lower supersaturations. In the case of the validation experiment with *c*_0_ = 16 mg/mL, the model returns a smoother concentration trajectory than the measured one, which has a relatively sharp decrease at approximately 200 min of residence time. This limitation of the model could similarly be due to imperfect model assumptions, which when coupled with a lack of *in situ* PAT make it challenging to identify the source of error in the model formulation.

The plots contain the simulated trajectories from the optimal parameter values and the quantified output uncertainty trajectories as shaded regions. It can be observed that the concentration uncertainty is not normally distributed, tending to be larger above the mean concentration trajectory rather than below in each of the simulated experiments. Since the solute concentration is the thermodynamic driving force of the crystallization process, these results suggest that faster kinetics found at higher concentrations lead to greater uncertainty than a system with lower supersaturation and slower kinetics. Additionally, the optimal parameter trajectories do not lie in the exact center of the quantified uncertainty trajectories, as propagation of the noncentered posteriors through the model also leads to noncentered output uncertainty. By tailoring the methodology to the model’s nonlinearity through *in silico* simulations and therefore avoiding linearization and simplifications to the methodology, the parametrized PBM’s predictive ability can be evaluated in much greater detail and confidence.

## 4 Conclusions

Effective parametrization of crystallization PBMs is impeded by measurement scarcity and the complex mathematical structure of the model, though these challenges can be tackled simultaneously via the integration of computational and experimental methods. The work presents an *in silico*/*in vitro* methodology for the parameter estimation and uncertainty quantification of an antisolvent batch protein crystallization system with sparse and offline measurements, tailored to the stiff and nonlinear PBM. Since the parameter estimation is restricted by the measurement scarcity, the evolution of first-, second-, and total-order sensitivity indices was used to explore each parameter’s identifiability during batch operation and suggest how to structure the *in vitro* measurement strategy. Approximate Bayesian Computation enabled the reconstruction of each parameter’s uncertainty distributions and then propagated to the PBM outputs via Monte Carlo simulations. Although the model’s reliability can still be improved with the introduction of more complex crystallization mechanisms, it remains a valuable tool to perform *in silico* simulations of lysozyme crystallization and examine sources of uncertainty. By avoiding simplifying assumptions and linearization techniques in the parameter estimation step, the PBM’s structure and parametrization can be assessed in much greater detail, as the insight recovered on parameter identifiability, posterior identification, and distribution of the output uncertainties would have been lost otherwise. Future work should integrate the extracted knowledge on parameter posteriors and output uncertainty to direct future experimentation and improve particle size predictions under the experimental limitations of the examined lysozyme crystallization system.

## Supplementary Material



## Notes

Further information on the global sensitivity analysis and Approximate Bayesian Computation with Differential Evolution methods used in this work and the experimental data measured and used for parameter estimation and model validation (concentration, crystal size distribution, quantiles, and respective variances) (PDF)

## Nomenclature

*A*_*g*_: Pre-exponential growth constant [m/s]
*A*_*j*_: Pre-exponential nucleation constant [ log # m^–3^ s^–1^]
*B*_0_: Nucleation rate [ log # m^–3^ s^–1^]
c: Solute concentration [mg/mL]
*D*_50_: 50% CSD quantile [μm]
*F*_*p*, *n*–*p*_^α^: Fisher distribution with confidence level α, number of parameters *p*, degrees of freedom *n* – *p* [−]
G: Growth rate [m/s]
g: Growth exponent [−]
*k*_*B*_: Boltzmann constant [J/K]
*k*_*v*_: Shape factor [−]
L: Crystal length [m]
*L_min_*, *L_max_*: Boundaries of the discretized length domain [m]
Δ*L*: Discretization bin width [m]
n: Crystal number density [# m^–1^ m^–3^]
N_conc.,e_: Number of concentration measurements in experiment *e* [−]
*N*_*exper.*_: Number of experiments [−]
p: Number of parameters to be estimated [−]
S: Supersaturation ratio [−]
*s*_*T*_: Total-order sensitivity index [−]
T: Temperature [K]
t: Time [s, min]
*v*_0_: Molecular volume [m^3^]
**Greek Letters**
α: Confidence level [−]
γ: Surface energy [J m^–2^]
δ: Dirac delta function [−]
θ: Vector of kinetic parameters [−]
ν: Total number of measurements [−]
ρ_*c*_: Crystal density [kg m^–3^]
σ_*conc.*,*D*_50__^2^: Measurement variance [mg/mL], [μm]
χ^2^: Chi-squared distribution [−]
