# Supplementary material for: Integrated In Vitro/In Silico Uncertainty Quantification Method for Protein Crystallization Models
Source: Ind Eng Chem Res. 2025 Jun 5;64(24):12025–35. doi: 10.1021/acs.iecr.4c04517 (PMC12186480; doi:10.1021/acs.iecr.4c04517)
Supplement: Supplementary file 1 [file ie4c04517_si_001.pdf]

Integrated *In Vitro/ In Silico* Uncertainty Quantification Method for Protein Crystallisation Models

Daniele Pessina[1,2], Jorge Calderon De Anda[3], Claire Heffernan[3], Jerry Y. Y. Heng[1,4], Maria M. Papathanasiou[1,2,*]

[1] Department of Chemical Engineering, Imperial College London, SW7 2AZ, UK

[2] The Sargent Centre for Process Systems Engineering, Imperial College London, SW7 2AZ, UK

[3] Chemical Development, Pharmaceutical Technology & Development, Operations, AstraZeneca, Macclesfield SK10 2NA, U.K

[4] Institute for Molecular Science and Engineering, Department of Chemical Engineering, Imperial College London, SW7 2AZ, UK

* maria.papathanasiou11@imperial.ac.uk

Supporting Information A: Details of Global Sensitivity Analysis

Variance-based Global Sensitivity Analysis, specifically the Sobol' method, is used in this work to examine the extent to which a change in model inputs, in this case kinetic parameters, affects the two model outputs, solute concentration and average particle size.

Defining $\boldsymbol{X} = (X_1, X_2, \ldots, X_k)$ as a vector of model inputs, the model response is defined as $Y = f(\boldsymbol{X})$. Reiterating the objective, the goal is to determine how each element of $\boldsymbol{X}$ contributes to changes in Y. Firstly, Analysis of Variance (ANOVA) decomposition is applied to Y (Eq 1) with the necessary orthogonality conditions (Eq 2 & 3).

$Y = f_0 + \sum_{i=1}^{k} f(X_i) + \sum_{i<j}^{k} f_{ij}(X_i, X_j) + \cdots + f_{1,2,\ldots,k}(X_1, X_2, \ldots, X_d)$	Eq 1
$\int_0^1 f_i(x_i)dx_i = 0$	Eq 2
$\int_0^1 f_{ij}(x_i, x_j)dx_i = \int_0^1 f_{ij}(x_i, x_j)dx_j = 0$	Eq 3

Due to the orthogonality conditions, $\mathrm{Var}(Y)$ is equal to the sum of individual variances, as shown in Eq. 4 and Eq. 5 with simplified terms V_i, V_{ij} and so forth for individual variances.

$\mathrm{Var}(Y) = \sum_{i=1}^{k} \mathrm{Var}\big(f_i(X_i)\big) + \sum_{1 \le i < j \le k} \mathrm{Var}\left(f_{ij}(X_i, X_j)\right) + \cdots + \mathrm{Var}\left(f_{1,2,\ldots,k}(X_i, X_2, \ldots, X_k)\right)$	Eq 4
$\mathrm{Var}(Y) = \sum_{i=1}^{k} V_i + \sum_{1 \le i < j \le k} V_{ij} + \cdots + V_{1,2,\ldots,k}$	Eq 5

The Sobol' sensitivity indices are defined from the ratios of variances, as shown in Eq 6-8, where $V_{\sim i}$ is the variance of all parameters except i.

$S_i = \frac{V_i}{\mathrm{Var}(Y)}$	Eq 6
$S_{ij} = \frac{V_{ij}}{\mathrm{Var}(Y)}$	Eq 7
$S_{Ti} = S_i + \sum_{j \neq i} S_{ij} + \cdots = 1 - \frac{V_{\sim i}}{\mathrm{Var}(Y)}$	Eq 8

The SobolHDMR software is used in this work to calculate the Sobol' sensitivity indices through a Random-Sampling High Dimensional Model Representation metamodel, which aims to represent the model response $Y = f(\boldsymbol{X})$ through an expansion of Legendre polynomials φ, truncated to orders t, l, l' (Eq. 9 & 10).[1]

$f_i(X_i) \approx \sum_{r=1}^{t} a_r^i \, \varphi_r(X_i)$	Eq 9
$f_{ij}(X_i, X_j) \approx \sum_{p=1}^{l} \sum_{q=1}^{l'} \beta_{pq}^{ij} \, \varphi_{pq}(X_i, X_j)$	Eq 10

By applying Parsival's theorem to the ANOVA decomposition, expressions for the first-, second- and total-order Sobol' sensitivity indices can be derived.

$$S_i \approx \frac{\sum_{r=1}^{t}\left(a_r^i\right)^2}{\mathrm{Var}(Y)}$$	Eq 11
$$S_{ij} \approx \frac{\sum_{p=1}^{l}\sum_{q=1}^{l'}\left(\beta_{pq}^{ij}\right)^2}{\mathrm{Var}(Y)}$$	Eq 12
$$S_{Ti} = S_i + \sum_{j \neq i} S_{ij} + \cdots = 1 - \frac{V_{\sim i}}{\mathrm{Var}(Y)}$$	Eq 13

Supporting Information B: Details of the Approximate Bayesian Computation method

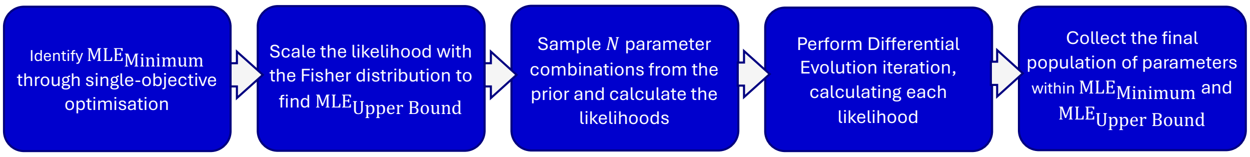

Figure SI 1: Workflow for the Approximate Bayesian Computation with Differential Evolution method

A workflow for the ABCDE method is presented in Figure SI 1. The standard ABC method relies on the assumption that the prior distribution resembles the posterior – if many of the drawn samples are found to be outside of the confidence region and are therefore rejected, the ABC step becomes computationally expensive. This challenge is exacerbated when ABC is applied to many-dimensional models, such as crystallisation PBMs, where the "curse of dimensionality" leads to many more rejected points, 'wasted' simulations and an overall inefficient method.[2] An ABC with differential evolution (ABCDE) method was proposed to address this limitation by incorporating a differential evolution (DE) genetic algorithm for the parameter distribution search.[3] In the first iteration, samples are drawn from the prior to form an initial parameter 'search-space' domain. From the second iteration, the ABCDE follows a modified DE algorithm with cross-over, mutation and an additional migration step. The latter is used to diversify the groups of candidate points and map the parameter posterior, without which the algorithm would simply find the global minimum MLE.

By replacing ABC's standard acceptance/rejection algorithm with a more-robust DE algorithm, any prior distribution composed of feasible parameters can be used, regardless of how similar it is to the parameter posterior. Rejected simulations are not wasted, but rather refined through DE generations to find the confidence region and fully cover it. Once the algorithm has converged, the final generation of candidates is statistically collected into parameter distributions.

Supporting Information C: Experimental Data used for Parameter Estimation and Model Validation

The experimental data used in this work is collected in Table SI 1.

Table SI 1 *- Experimental Data used for Parameter Estimation*

Experimental data used for Parameter Estimation		
**Time - min**	**Measured concentration [mg/ml]**	**Concentration Variance**
**Estimation Experiment 1**		
0	18.739	0.126
25	18.993	0.185
50	9.766	1.217
80	7.965	0.855
130	7.044	0.339
175	6.804	0.072
245	5.898	0.145
305	5.709	0.137
Measured $D_{50}[\mu\mathrm{m}]$	10.731	5.414
**Estimation Experiment 2**		
0	17.766	0.005
70	17.333	0.029
90	13.802	6.113
130	11.488	4.820
180	9.512	0.501
230	8.229	0.223
295	7.758	0.792
365	7.513	0.479
410	7.283	0.383
Measured $D_{50}[\mu\mathrm{m}]$	9.387	3.953
**Estimation Experiment 3**		
0	15.032	0.148
30	15.060	0.120
80	15.060	0.137
120	14.596	0.075
165	13.403	1.707
295	10.145	1.850
350	9.514	0.592
400	9.040	0.351
Measured $D_{50}[\mu\mathrm{m}]$	7.259	1.033

Experimental data used for Model Validation		
**Time - min**	**Measured concentration [mg/ml]**	**Concentration Variance**
**Validation Experiment 1**		
**0**	**19.738**	**-**
**35**	**18.580**	**-**
**60**	**10.552**	**-**
**90**	**8.698**	**-**
**125**	**8.196**	**-**
**165**	**6.361**	**-**
**225**	**5.180**	**-**
**260**	**4.934**	**-**
**315**	**4.083**	**-**
**Measured $D_{50}[\mu\text{m}]$**	**11.127**	**-**
**Validation Experiment 2**		
**0**	**16.139**	**-**
**35**	**16.389**	**-**
**70**	**16.344**	**-**
**100**	**16.301**	**-**
**130**	**16.013**	**-**
**155**	**16.013**	**-**
**180**	**14.743**	**-**
**230**	**10.591**	**-**
**275**	**9.804**	**-**
**330**	**8.001**	**-**
**390**	**7.522**	**-**
**Measured $D_{50}[\mu\text{m}]$**	**13.733**	**-**

Supporting Information D: Measured and predicted crystal size distributions

The experimentally measured crystal size distributions, along with the predicted size distribution of the optimal vector of parameters, have been collected in

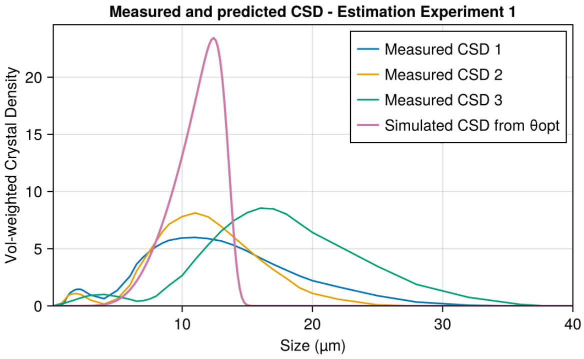

Figure SI 2: Measured and predicted crystal size distribution for Estimation Experiment

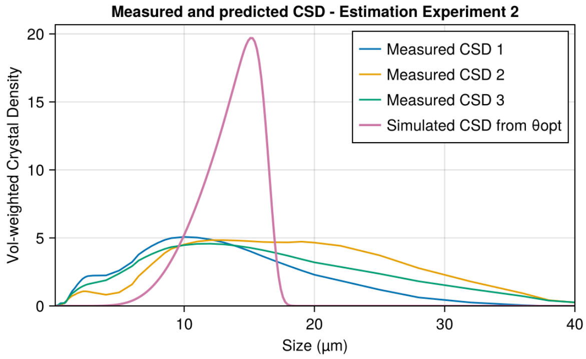

Figure SI 3: Measured and predicted crystal size distribution for Estimation Experiment 2

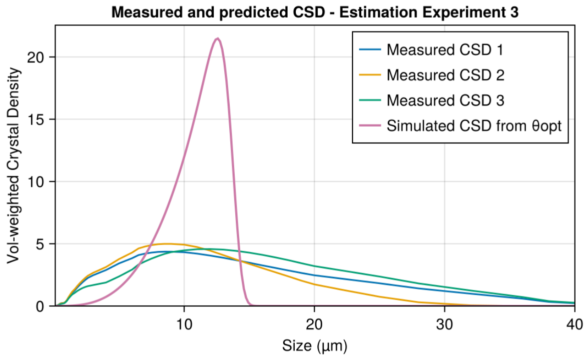

Figure SI 4:: Measured and predicted crystal size distribution for Estimation Experiment 3

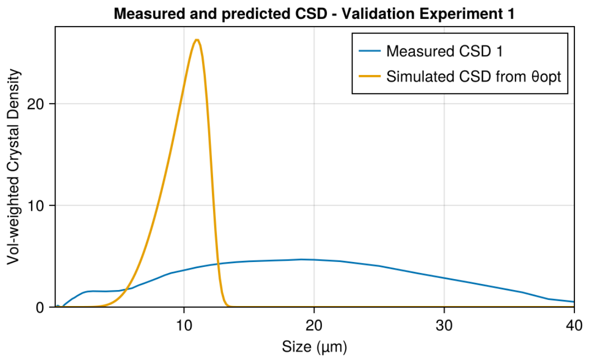

Figure SI 5: Measured and predicted crystal size distribution for Validation Experiment 1

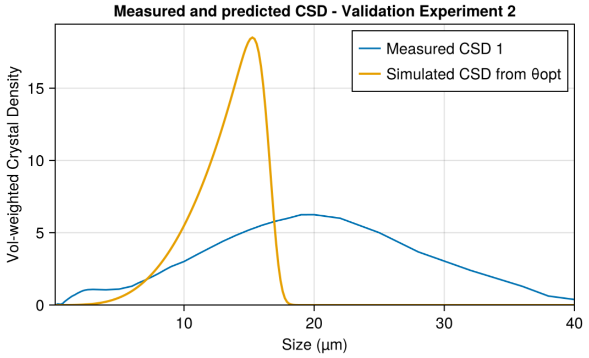

Figure SI 6: Measured and predicted crystal size distribution for Validation Experiment 2

Supporting Information: References

(1) Kucherenko, S. SOBOLHDMR: A General-Purpose Modeling Software. In *Synthetic Biology*; Polizzi, K. M., Kontoravdi, C., Eds.; Humana Press: Totowa, NJ, 2013; pp 191–224. https://doi.org/10.1007/978-1-62703-625-2_16.

(2) Beaumont, M. A. Approximate Bayesian Computation in Evolution and Ecology. *Annual Review of Ecology, Evolution, and Systematics* **2010**, *41*, 379–406.

(3) Turner, B. M.; Sederberg, P. B. Approximate Bayesian Computation with Differential Evolution. *Journal of Mathematical Psychology* **2012**, *56* (5), 375–385. https://doi.org/10.1016/j.jmp.2012.06.004.
